# Progress on Omega-3 fatty acids for the comprehensive and targeted treatment of spinal cord injury

**DOI:** 10.1038/s41413-025-00461-w

**Published:** 2026-01-12

**Authors:** Zhongze Yuan, Lusen Shi, Xiao-Na Tao, Xiangchuang Fan, Han Zheng, Yifan Shang, Xiaoqing Zhao, Fan Yang, Hui Lin, Peng Xiao, Bo Chu, Jichuan Qiu, Shaohui Zong, Ning Ran, Xiaohong Kong, Jin-Peng Sun, Hengxing Zhou, Shiqing Feng

**Affiliations:** 1 The Second Hospital of Shandong University, Cheeloo College of Medicine, Shandong University, Jinan, Shandong China; 2 Department of Orthopaedics, Qilu Hospital of Shandong University, Shandong University Centre for Orthopaedics, Advanced Medical Research Institute, Cheeloo College of Medicine, Shandong University, Jinan, Shandong China; 3 Key Laboratory Experimental Teratology of the Ministry of Education and Department of Biochemistry and Molecular Biology, School of Basic Medical Sciences, Cheeloo College of Medicine, Shandong University, Jinan, Shandong China; 4 State Key Laboratory of Crystal Materials, Shandong University, Jinan, Shandong China; 5 Department of Spine Osteopathia, The First Affiliated Hospital of Guangxi Medical University, Nanning, Guangxi China; 6 Wuming Hospital of Guangxi Medical University, Nanning, Guangxi China; 7 Advanced Medical Research Institute, Cheeloo College of Medicine, Shandong University, Jinan, Shandong China; 8 Department of Orthopaedics, Tianjin Medical University General Hospital, International Science and Technology Cooperation Base of Spinal Cord Injury, Tianjin Key Laboratory of Spine and Spinal Cord, Tianjin, China

**Keywords:** Diseases, Physiology

## Abstract

Traumatic spinal cord injury (SCI) is a debilitating condition characterized by the impairment of neural circuits, leading to the loss of motor and sensory functions and accompanied by severe complications. Substantial research has reported the therapeutic potential of Omega-3 fatty acids for the central nervous system, particularly after traumatic SCI. Omega-3 fatty acids may contribute to improving SCI recovery through their anti-inflammatory, anti-oxidative, neurotrophic, and membrane integrity-preserving properties. These functions of Omega-3 fatty acids are primarily mediated via the activation of G protein-coupled receptor 120 (GPR120), commonly known as the fish oil-specific receptor. Advancements in understanding of the molecular mechanisms of GPR120’s recognition of Omega-3 fatty acids and its downstream signaling mechanisms has significantly promoted research on the pharmacological potential of Omega-3 fatty acids and the development of highly selective and high-affinity alternatives. This review aims to provide in-depth analysis of the comprehensive therapeutic potential of Omega-3 fatty acids for SCI and its accompanying complications, and the prospects for developing novel drugs based on the recognition of Omega-3 fatty acids by GPR120.

## Introduction

Traumatic spinal cord injury (SCI) is a debilitating neurological condition characterized by the disruption of neural communication between the brain and effector organs, as well as the interruption of autonomic nerve circuits.^1–3^ This condition is often accompanied by severe complications such as neuropathic pain, osteoporosis, and obesity.^2,4^ Traumatic SCI has a significant prevalence and is associated with a high disability rate, significantly impacting the quality of patients’ lives and imposing substantial economic burdens on individuals and society.^1,5,6^ Therefore, there is a critical importance to identify potential clinical intervention strategies for SCI. Current promising clinical and preclinical treatments for SCI include surgical decompression, pharmacotherapy, cell and biological agents, biological carrier transplantation, epidural electrical stimulation, and brain-spinal cord-computer interfaces.^1,7–13^ Pharmacotherapy represents a pivotal approach to effectively address the challenges associated with SCI, due to its convenient industrialized production, wide dissemination, minimal requirement for medical guidance, and affordability. However, the limited effectiveness and adverse effects of current pharmacotherapies hinder their widespread clinical application, leading to a lack of suitable pharmacological interventions for the clinical management of SCI. In detail, pharmacological strategies can be categorized into two major classes: microenvironmental regulators, which target the inflammatory and inhibitory microenvironment; and neuroregenerative activators, which target the neurotrophic microenvironment and directly promote the reconstruction of neural circuits.^1^ Among the pharmacological agents that have entered clinical trials, each possesses its own limitations. Corticosteroids were among the earliest agents to be applied in clinical settings; however, their therapeutic efficacy has been limited due to a narrow treatment window (being effective only when administered within 3–8 h post-injury) and the potential adverse effects associated with high-dose administration.^14–16^ Minocycline and ganglioside have shown promising therapeutic effects in both animal studies and early-phase clinical trials (Phase I/II); however, they failed to demonstrate efficacy in Phase III clinical trials.^17,18^ Riluzole and neurotrophins, on the other hand, are associated with unexpected side effects, including locomotor ataxia and lethargy, as well as nociceptive sprouting accompanied by neuropathic pain, respectively.^19,20^ Consequently, there is an urgent need to develop affordable medicines that leverage novel therapeutic targets to enhance treatment outcomes for SCI.

G protein-coupled receptors (GPCRs) constitute the largest family of membrane-protein, widely distributed throughout the human body.^21^ GPCRs act as pivotal hubs for signal transmission and respond to a variety of signals, including hormones, neurotransmitters, ions, photons, odorants, and other stimuli.^21^ GPCRs mediate various signaling pathways, contributing to numerous physiological and pathological functions.^21^ They are thus recognized as significant drug targets, as recent studies indicate that ~34%–36% of drugs approved by the United States Food and Drug Administration specifically target 108 distinct GPCR types, with an additional 66 potential GPCR targets in development.^22,23^ GPCR-targeting medicines were initially designed for the treatment of various diseases, and their application has expanded to the treatment of central nervous system (CNS) disorders, including Alzheimer’s disease and multiple sclerosis.^23^ These advancements have spurred interest in exploring GPCRs and their small molecule ligands in the pathological processes and treatment of SCI, offering prospects for comprehensive and targeted drug therapies.

Omega-3 fatty acids, essential nutrients that cannot be synthesized endogenously, are primarily derived from cold-water fish oil.^24^ Omega-3 fatty acids possess multiple beneficial effects on the human body, including the regulation of glucose and lipid metabolism, and anti-inflammatory and anti-oxidative properties, neurotrophic effects, and membrane integrity preservation.^25–27^ The specific binding receptor for Omega-3 fatty acids, G protein-coupled receptor 120 (GPR120), is integral to these molecular pathways and is pivotal for their therapeutic potential.^28–30^ Over the past 50 years, interest in Omega-3 fatty acids for treating CNS disorders and traumatic injuries has significantly increased.^31,32^ Consumption of fish oil supplements prior to illness onset has shown preventive effects, particularly when administered as a nutritional intervention to alleviate systemic inflammation after trauma.^32–37^

A primary challenge in Omega-3 fatty acids therapy is low efficacy, even at high doses.^38^ Recent clinical trials exploring allosteric modulators and biased agonists offer promising avenues for more effective GPCR targeting and activation.^23,28,38^ A recent study provides promising evidence regarding the capability of GPR120 to discern distinct double bond positions in fatty acids, subsequently triggering downstream effectors.^39^ This discovery provides a theoretical basis for developing potent pharmaceutical agents specifically targeting GPR120.

This review provides a thorough description of the wide-ranging and specific effects of Omega-3 fatty acids relating to SCI and associated complications, alongside details of the recent advancements in the recognition of Omega-3 fatty acids double bonds by GPR120. The findings propose a novel direction—developing highly selective and high-affinity Omega-3 fatty acids substitutes that target distinct downstream effectors of GPR120; These substitutes hold substantial potential to enhance drug efficacy and reduce adverse effects, ultimately improving conprehensive and targeted clinical treatments for SCI.

## Omega-3 fatty acids

### Fatty acids

Fatty acids are organic compounds that serve as important components of lipids.^40^ Based on their length, fatty acids can be categorized as short-chain (fewer than six carbon atoms), medium-chain (six to twelve carbons), and long-chain (twelve or more carbons).^41^ According to their degree of saturation, fatty acids can be classified as saturated, monounsaturated, or polyunsaturated. Saturated fatty acids have no double bonds between their carbon atoms, while monounsaturated fatty acids have one double bond, and polyunsaturated fatty acids have two or more double bonds.^42^ The classification and properties of fatty acids have been extensively studied due to their significant implications for human health, particularly Omega-3 fatty acids, which can not only exhibit antioxidant and anti-inflammatory effects but also regulate platelet homeostasis and lower the risk of thrombosis.^43^

### Polyunsaturated fatty acids

Polyunsaturated fatty acids can be further categorized into Omega-3 and Omega-6 fatty acids based on the position of the first double bond from the methyl end of the fatty acids. Omega-3 fatty acids have a double bond at the third carbon atom from the end of the carbon chain, whereas Omega-6 fatty acids have a double bond at the sixth carbon atom.^42^ Omega-3 fatty acids, known as essential fatty acids, include eicosapentaenoic acid (EPA), docosahexaenoic acid (DHA), alpha-linolenic acid (ALA), and docosapentaenoic acid (DPA).^44^ EPA and DHA are primarily found in cold-water fish.^45^ DPA is also widely found in marine foods, but generally at lower levels compared to EPA and DHA.^46^ ALA is predominantly found in plant-based foods, including flaxseed, echium seeds, and walnuts.^47^ Arachidonic acid (AA), a form of Omega-6 fatty acid, is mainly found in low-plant species like mosses and lichens.^48^ The structural dissimilarities of these acids lead to functional differences in terms of their effects on inflammation and metabolism.^49^ Omega-3 fatty acids play significant roles in preventing and treating of inflammatory diseases and metabolic disorders by inhibiting the activation of NOD-like receptor thermal protein domain associated protein 3 (NLRP3) and reducing the secretion of inflammatory factors.^29,50^ In contrast, Omega-6 fatty acids exhibit pro-inflammatory properties by increasing the production of inflammatory leukotrienes (LT), prostaglandins (PG), and cytokines.^51^ Therefore, a well-balanced Omega-3/Omega-6 ratio is crucial for preventing inflammatory and metabolic diseases^52^ (Table 1).

**Table 1:** The structure and functions of polyunsaturated fatty acids and their metabolites

Classification	Structure	Key Functions	Pharmaceutical Applications	Reference
Omega-3 fatty acids				
α-Linolenic Acid (ALA)		- Serves as a precursor of EPA and DHA - Lowers blood pressure and cardiovascular risks - Enhances immune function and modulates lipid metabolism	- Prevention of atherosclerosis and hypertension - Dietary supplementation for metabolic syndrome	^273^
Eicosapentaenoic Acid (EPA)		- Lowers triglycerides and inflammation. - Exerts anti-thrombotic effects by inhibiting platelet aggregation	- Cardiovascular drugs (e.g., Vascepa®) - Adjuvant therapy for depression and autoimmune diseases	^274^
Docosahexaenoic Acid (DHA)		- Critical for brain and retinal development - Provides neuroprotective effects against Alzheimer’s disease - Improves synaptic plasticity and memory	- Infant formula additives for cognitive development - Neurodegenerative disease treatment (e.g., dementia)	^275^
Omega-6 fatty acids				
Linoleic Acid (LA)		- Serves as an essential fatty acid for skin barrier function - Reduces LDL cholesterol and prevents atherosclerosis	- Treatment of eczema and dermatitis - Formulation of lipid-lowering drugs	^276^
Arachidonic Acid (AA)		- Serves as a precursor of pro-inflammatory eicosanoids (e.g., prostaglandins) - Regulates neuronal signaling and synaptic plasticity	- Anti-cancer drug development (e.g., selective cytotoxicity in tumor cells) - Neuroinflammatory disease research	^277^
γ-Linolenic Acid (GLA)		- Acts as an anti-inflammatory agent by converting to PGE1 - Reduces symptoms of rheumatoid arthritis and diabetic neuropathy	- Nutritional supplements for autoimmune disorders - Topical formulations for skin health	^278^
Metabolites of Omega-3 fatty acids				
Neuroprotectin D1 (NPD1)		- Protects retinal cells and neurons from oxidative stress - Modulates anti-apoptotic pathways in Alzheimer’s disease	- Therapeutic agent for neurodegenerative diseases - Ophthalmic applications for xerophthalmia and retinopathy	^279^
Resolvin E1 (RvE1)		- Acts as a potent anti-inflammatory and pro-resolving mediator - Reduces neutrophil infiltration and promotes macrophage phagocytosis - Enhances tissue repair and regeneration	- Treatment of chronic inflammatory diseases (e.g., rheumatoid arthritis, periodontitis) - Wound healing and tissue regeneration therapies	^140^
Resolvin E2 (RvE2)		- Modulates leukocyte migration and cytokine production - Reduces vascular inflammation and endothelial activation	- Potential therapy for atherosclerosis and vascular inflammation	^280^
Resolvin D1 (RvD1)		- Promotes resolution of inflammation and tissue homeostasis - Reduces pro-inflammatory cytokines and enhances efferocytosis - Protects against neuroinflammation and sepsis	- Neuroprotective agent for Alzheimer’s and Parkinson’s diseases - Treatment for sepsis and acute lung injury	^281^
Resolvin D2 (RvD2)		- Enhances bacterial clearance and reduces systemic inflammation - Regulates neutrophil trafficking and promotes tissue repair	- Therapeutic for bacterial infections and sepsis - Wound healing and post-surgical recovery	^282^
Resolvin D3 (RvD3)		- Modulates immune cell responses and reduces chronic inflammation - Promotes resolution of allergic airway inflammation	- Treatment of asthma and chronic obstructive pulmonary disease (COPD)	^283^
Resolvin D4 (RvD4)		- Reduces vascular inflammation and promotes endothelial barrier function - Enhances resolution of acute kidney injury	- Potential therapeutic for acute kidney injury and cardiovascular diseases	^281^
Resolvin D5 (RvD5)		- Exerts anti-inflammatory and pro-resolving effects in metabolic disorders- Reduces adipose tissue inflammation and improves insulin sensitivity	- Treatment of obesity-related metabolic syndrome and type 2 diabetes	^284^

### Overview of GPR120

Omega-3 fatty acids are recognized by GPR120, a receptor that plays a crucial role in regulating metabolic and immune functions.^53^ Studies have demonstrated an increase in the expression of GPR120 after SCI, with the most significant increase observed in astrocytes.^54^ Further studies revealed that GPR120 can regulate astrocyte proliferation by influencing the cell cycle,^54^ suggesting that GPR120 is a potential therapeutic target for the treatment of SCI. Current investigations on GPR120 primarily focuses on its regulatory roles in both physiological and pathological contexts.^55–57^ Further research is needed on GPR120’s intricate signal transduction mechanisms in order to develop safe and effective GPR120-targeted drugs for clinical use.

## Basic and clinical studies on the effects of Omega-3 fatty acids on spinal cord injury

Following SCI, various microenvironmental alterations, including pro-inflammatory microglial responses, excessive reactive oxygen species (ROS) that drive oxidative stress, and astrocyte proliferation resulting in fibrotic scar—collectively hinder neurological recovery and complicate SCI rehabilitation. Omega-3 fatty acids, a class of polyunsaturated fatty acids chiefly consisting of eicosapentaenoic acid (EPA), docosahexaenoic acid (DHA), and alpha-linolenic acid (ALA), serve multifaceted roles in enhancing microenvironment, partly by targeting a series of molecules such as GPR120. These roles include anti-inflammatory, antioxidant, neurotrophic, and cell membrane integrity-preserving functions. Thus, Omega-3 fatty acids hold promising potential in modulating central nervous system inflammation, particularly in the treatment of acute neurological damage caused by traumatic events. Overall, Omega-3 fatty acids demonstrate broad therapeutic potential in the field of spinal cord injury treatment. However, further research is required to fully elucidate their mechanisms of action and applicability to determine the optimal therapeutic strategies.

### Anti-inflammation

Inflammation is a pathophysiological mechanism initiated by microglia and peripherally derived myeloid cells following SCI and plays a dual role in the repair process of the injured spinal cord.^58,59^ Microglia and macrophages not only mitigate the inhibitory microenvironment post-SCI by clearing apoptotic cellular debris,^60,61^ but also can facilitate wound sealing to limit injury expansion and debris removal, thereby potentially contributing to the restoration of motor function.^60,62–64^ Meanwhile, limiting the harmful pro-inflammatory effects of microglia and macrophages is pivotal for SCI treatment. During the acute phase of SCI, initial mechanical tissue damage triggers damage-associated molecular patterns (DAMPs).^65,66^ These DAMPs activate microglia—resident CNS surveillance cells—resulting in robust inflammation and oxidative stress.^65,67^ A variety of chemokines—released by astrocytes, resident microglia, and endothelial cells—are subsequently up-regulated in spinal cord. These chemokines, in turn, recruit neutrophils and monocytes derived from peripheral blood and the hematopoietic system to traverse the damaged blood-spinal cord barrier. Some of these exogenous myeloid cells and activated microglia release pro-inflammatory factors, proteases, and other cytotoxic factors.^1^ The inflammatory microenvironment formed by these compounds substantially contributes to the secondary injury, resulting in neuron and oligodendrocyte death and promoting glial scar formation.^68,69^ Thus, reducing inflammation from activated microglia, macrophages, and neutrophils while improving the inflammatory microenvironment are effective strategies to alleviate neurological dysfunction after SCI.

The anti-inflammatory properties of Omega-3 fatty acids first identified in cardiovascular diseases.^70^ Given their efficacy, researchers have advocated for their use in other conditions characterized by acute and chronic inflammation.^31,71–74^ The role of Omega-3 fatty acids in regulating neuroinflammation has been extensively investigated in recent decades.^75,76^ Chronic inflammation of the brain is primarily characterized by overactivation of microglia and the release of inflammatory factors, which are viable targets for Omega-3 fatty acids.^77^ Knockout of Acsl6(Acyl-CoA Synthetase Long-Chain Family Member 6), a gene associated with Omega-3 fatty acids synthesis in neurons, influences age-related inflammation, thereby affecting motor function and memory.^78^ Pretreatment with DHA reverses TNFα-induced neuroinflammation in hypothalamic neurons via GPR120, while decreased endogenous GPR120 expression impairs Omega-3 fatty acids efficacy.^79^ In vitro studies indicate that DHA can reduce inflammatory biomarkers in microglia and hippocampal neurons, underpinning its potential for treating cognitive decline and depressive symptoms.^77,80,81^ Notably, a large-scale multi-center study has revealed that Omega-3 fatty acids supplementation alleviates primary injury effects, secondary intracellular metabolic disorders, and persistent inflammation associated with traumatic brain injury.^82^ Furthermore, such supplementation has demonstrated the ability to improve neurological recovery post-cerebral ischemia by modulating microglia polarization.^83^ These findings highlight the significant prospective role of Omega-3 fatty acids in regulating CNS inflammation, particularly in the treatment of acute nerve injury resulting from traumatic events.

Given that multiple CNS disorders share common pathological mechanisms, it is plausible to extend the potential therapeutic utility of Omega-3 fatty acids to SCI treatment. At the molecular level in an SCI model, Omega-3 fatty acids have been shown to significantly inhibit the assembly and activation of the NOD-like receptor thermal protein domain associated protein 1 (NLRP1) and NLRP3 inflammasome, reducing IL-1, IL-6, IL-18, and TNFα levels.^84–86^ At the cellular level in an SCI model, Omega-3 fatty acids have been found to reduce the number of, and inhibit activation in, microglia and exogenous monocytes.^87,88^ Further studies indicate that Omega-3 fatty acids can reduce microglia aggregation^89^ and inhibit microglia activation.^90^ Fat-1 transgenic mice, which carry the Fat-1 gene from *Caenorhabditis elegans*, can insert a double bond into an unsaturated fatty-acid hydrocarbon chain and thus convert Omega-6 to Omega-3 fatty acids.^91^ These mice therefore serve as an effective model to investigate Omega-3 and Omega-6 fatty acids dynamics. In an SCI model employing Fat-1 mice, investigators observed reduced inflammatory cell activation, lower production of inflammatory factors, and improved motor function recovery.^92^

**Fig. 1:**
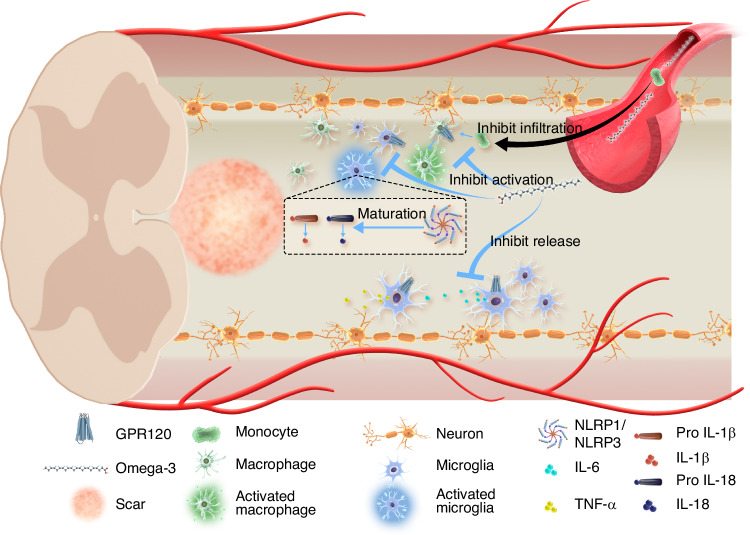
Overview of Omega-3 fatty acids facilitating the repair of spinal cord injury through anti-inflammatory properties. Omega-3 fatty acids can access the spinal cord injury microenvironment by penetrating the blood-spinal cord barrier. Omega-3 fatty acids suppress monocyte infiltration from the bloodstream and reduce the activation of macrophages and microglia. Regarding the regulation of inflammatory factors, Omega-3 fatty acids inhibit the release of IL-6 and TNF-α from microglia and block the maturation of IL-1β and IL-18 precursors by modulating the pathway of the NLRP1/NLRP3 inflammasome, thus reducing their level in the spinal cord lesion

Following SCI, significant alterations in membrane remodeling-related metabolites occurred, including the up-regulation of the anti-inflammatory Omega-3 fatty acids DHA and DPA.^93^ Metabolomic analyses indicate that elevated blood concentrations of DHA and DPA negatively correlate with BBB(Basso-Beattie-Bresnahan) scores. This implies that Omega-3 fatty acids may serve as potential biochemical markers and promoters of locomotor recovery in SCI mice.^93,94^ This correlation was further supported by López-Vales et al.,^95^ who found that fenretinide, an anticancer agent that elevates the Omega-3/Omega-6 ratio, exhibits significant inhibitory effects on neuroinflammation by decreasing levels of AA and increasing those of DHA in both serum and spinal cord tissue. Additionally, in patients with chronic SCI, 3 months of dietary Omega-3 fatty acids supplementation reduced chronic inflammation, suppressing cytokines such as IL-1β, IL-6, TNF-α, and interferon-gamma.^96^ However, another clinical study found that Omega-3 fatty acids mainly inhibited inflammation during the acute phase, with minimal effects on chronic inflammation.^97^ In summary, numerous pre-clinical and clinical studies support the capacity of Omega-3 fatty acids to inhibit neuroinflammation in acute and, potentially, chronic stages of SCI. Nevertheless, clinical trials involving Omega-3 fatty acids have not yet produced consistent results (Fig. 1).

In summary, Omega-3 fatty acids show substantial potential to enhance SCI outcomes via their anti-inflammatory effects. Nonetheless, the precise mechanisms by which they modulate the inflammatory microenvironment in immune cells post-SCI require further clarification.

### Anti-oxidation

Oxidative stress arises from an imbalance in the cellular redox state triggered by excessive reactive oxygen species (ROS).^98^ Oxidative stress is integral to the secondary injury cascade in SCI, culminating in neuronal apoptosis and autophagy.^99^ Inflammation and oxidative stress frequently coexist, operating via a reciprocal feedback loop.^99,100^ Oxidative stress leads to inflammation through nuclear factor kappa-B (NF-κB) and other pathways, in turn, inflammation is associated with the induction of intracellular oxidative stress in mitochondria and endoplasmic reticulum.^101–104^ Given the fact that oxidative stress and inflammation can be reactivated via compensatory pathways resulting in treatment failure, some studies have proposed a dual therapeutic anti-oxidant and anti-inflammatory effect of Omega-3 fatty acids on acute SCI.^85,95,105^ These dual properties also act synergistically.^99^ For instance, King et al.^106^ and Huang et al.^87^ report that intravenous injection of DHA, 30 min post-SCI, effectively suppressed cellular oxidative stress and minimized the extent of the injury area through the reduction of lipid peroxidation, protein oxidation, RNA/DNA oxidation, and cyclooxygenase-2 (COX-2) levels. Additionally, apart from the direct regulation of acute SCI oxidative stress through intravenous administration, a long-term preventive Omega-3 fatty acids-rich diet was shown in the rat to enhance anti-oxidant defense by maintaining metabolic homeostasis after SCI.^107^ A recent study indicates that Omega-3 fatty acids lessen endoplasmic reticulum stress-induced neuroinflammation after SCI by inhibiting histone deacetylase 3, thus advancing neurological functional recovery.^108^ Considering the interconnected molecular pathways, the administration of anti-oxidant and anti-inflammatory treatments with the same medication may lead to improved comprehensive efficacy.^105^ Therefore, Omega-3 fatty acids present significant potential for enhancing SCI outcomes due to these antioxidant and combined effects. Further elucidation of common anti-inflammatory and anti-oxidant pathways such as erythroid 2-Related Factor 2 and NF-κB would be advantageous for exploring the potential of Omega-3 fatty acids.

### Regeneration of neural circuits

Activated microglia and macrophages migrate toward the SCI site, leading to glial hyperplasia, extracellular matrix remodeling, and the formation of a fibrous scar within the lesion.^109^ Reactive astrocytes, oligodendrocyte precursors, and microglia also contribute to the formation of glial scars surrounding the fibrous scar.^109^ The glial scar border forms to segregate the neural lesion and to isolate spreading inflammation, ROS, and excitotoxicity at the injury epicenter to preserve surrounding healthy tissue.^110^ While this physiological response preserves viable neural tissue, it is also detrimental to axon sprouting and neural regeneration.^109,110^ The presence of chondroitin sulfate proteoglycans (CSPGs) and Nogo-A around the glial scar poses a significant challenge to the regeneration of nerve axons and synapses.^1^ In the spinal cord, the myelin sheath is formed by oligodendrocytes that wrap around nerve axons, aiding in the conduction of nerve impulses.^111^ After SCI, the apoptosis of oligodendrocytes leads to demyelination of neurons.^112^ Reducing the damage to myelin, removing damaged myelin debris, and promoting remyelination are potential approaches thereby safeguarding adjacent neurological function.^113,114^

**Fig. 2:**
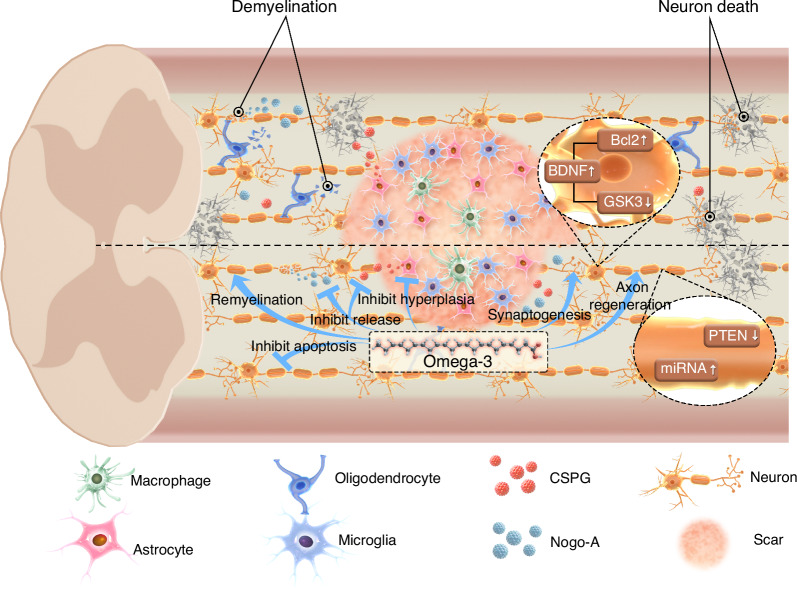
Overview of Omega-3 fatty acids facilitating the repair of spinal cord injury by the regeneration of neural circuits. In spinal cord injury, the destruction of neural circuits is accompanied by the aggregation of astrocytes, microglia, and macrophages at the site of injury, resulting in glial scar formation. Oligodendrocyte apoptosis leads to demyelination in functional neurons, while neuronal death directly contributes to further neural circuit disruption. Omega-3 fatty acids mitigate the pathological damage through multiple mechanisms: ①Preventing oligodendrocyte apoptosis and neuronal demyelination by inhibiting inflammation; ②Inhibiting the release of chondroitin sulfate proteoglycans (CSPGs) and Nogo-A, thereby improving axon regeneration; ③Reducing GFAP expression and astrocyte hyperplasia to minimize dense glial scar formation; ④Promoting the production of brain-derived neurotrophic factor (BDNF) which regulates apoptosis through GSK-3 and Bcl-2 pathways; ⑤Upregulating miRNA-21 while downregulating PTEN to improve synaptogenesis

The direct effect of Omega-3 fatty acids on neural circuits that promote regeneration has been observed in various neurodegenerative and nerve injury models.^83^ The efficacy of these fatty acids in promoting nerve restoration has also been demonstrated in studies involving corneal nerve injury,^115^ depression,^116^ and other conditions, where a high Omega-3 fatty acids diet or local administration has yielded favorable outcomes. Within the complex microenvironment of SCI, Omega-3 fatty acids exhibit the capacity to target glial scars and neurons, thereby facilitating the reconstruction of neural circuits.^86,117^ The molecular mechanism underlying the reduction of scar formation involves the inhibition of astrocyte hyperplasia^118,119^; Omega-3 fatty acids can reduce the expression of the astrocyte hyperplasia marker, glial fibrillary acidic protein (GFAP), thereby inhibiting scar formation.^86^ One of the most widely studied molecular mechanisms underlying the neurotrophic effects of Omega-3 fatty acids is the promotion of brain-derived neurotrophic factor (BDNF) production.^120,121^ BDNF plays a crucial role in neuroprotection and axonal growth in SCI and it regulates neuron apoptosis through glycogen synthase kinase-3 (GSK-3) and B-cell lymphoma-2 (Bcl-2).^122^ In a model of chronic spinal cord compression injury, dietary administration of low doses of Omega-3 fatty acids combined with curcumin significantly increased the BDNF content in the injured spinal cord, compared to a Western diet high in saturated fats.^123^ Omega-3 fatty acids have the potential to enhance neuroplasticity, promote synaptogenesis, and germination of the intact corticospinal tract, particularly 5-hydroxytryptamine fibers, when combined with rehabilitative exercise.^117^ The underlying mechanisms include the upregulation of miRNA-21 and phosphorylated protein kinase B(AKT) and the downregulation of phosphatase and tensin homolog (PTEN).^124^ Omega-3 fatty acids have been shown to prevent neuron demyelination by inhibiting oligodendrocyte apoptosis.^84^ However, it is important to note that phagocytosis of myelin debris by activated macrophages plays a crucial role in improving the SCI microenvironment^114^ (Fig. 2). These factors must be considered when balancing the effects of Omega-3 fatty acids in inhibiting oligodendrocyte apoptosis and with the potential downside of suppressing macrophage activation, which can ultimately reduce the clearance of harmful myelin debris. To optimize treatment strategies, determining the appropriate dosage is essential while considering both short-term and long-term biological effects.

### Other mechanisms

Polyunsaturated fatty acids predominantly reside within the membrane structure of human cells, playing a vital role in stabilizing the membrane, supporting the repair of damaged plasma membranes, facilitating the healing of damaged plasma membranes and modulating ion channels.^125,126^ Research has demonstrated that Omega-3 fatty acids help preserve membrane fluidity in the brain, which is essential for normal neuromotor and cognitive function.^104,127^ At the chronic stage of SCI, a diet high in Omega-3 fatty acids and curcumin led to a significant increase in syntaxin-3 and a reduction in 4-hydroxynonenal levels in the lumbar enlargement, indicating a possible healing effect on the plasma membrane.^123^ The integration of Omega-3 fatty acids into cell membranes affects signal transduction.^128^ Omega-3 fatty acids can inhibit neuronal depolarization by blocking voltage-sensitive calcium and sodium channels while activating double-pore potassium channels, thereby reducing in the excitability of hippocampal and spinal cord neurons, and mitigating neurotoxicity caused by the outward flow of glutamic acid.^129,130^ Ion channel regulation in the nervous system may be a key mechanism by which fatty acids influence SCI, suggesting that an electrophysiological perspective as a viable approach for addressing SCI.

Omega-3 fatty acids have been shown to disrupt lipid raft domains, which are cholesterol- and phospholipid-rich microenvironments in cell membranes, thereby altering the function and distribution of proteins residing in these domains.^131,132^ Toll-like receptor 4 (TLR4) is important in receiving various pro-inflammatory signals, such as DAMPs and pathogen-associated molecular patterns (PAMPs), following CNS injury.^133^ DHA inhibits the translocation of TLR4 to the cell membrane, thereby mitigating the inflammatory response of microglia.^134^

Resolvins D1 and D2 have been demonstrated to inhibit the activation of the Transient Receptor Potential Vanilloid 1 (TRPV1) and Ankyrin 1 (TRPA1) ion channels by perturbing the integrity of lipid rafts in the plasma membranes of sensory neurons. This disruption leads to a modulation of the receptors’ electrical potential, which in turn facilitates peripheral analgesia.^135^

The therapeutic efficacy of Omega-3 fatty acids in SCI extends beyond the fatty acids themselves to include beneficial effects mediated by their metabolites and synthetic derivatives.^136^ Specifically, supplementation with synaptic amide, a molecule derived from DHA, has been shown to enhance axonal growth and synaptogenesis, while reducing a reduction in inflammatory responses.^137,138^ These effects are mediated by GPR110, a receptor distinct from GPR120, which is targeted by Omega-3 fatty acids.^139^ Resolvin is an endogenous lipid mediator generated from Omega-3 fatty acids and its metabolite.^140^ In addition to their potent anti-inflammatory effects, resolvins also modulate leukocyte trafficking and enhance the non-inflammatory phagocytosis of apoptotic neutrophils by macrophages, thereby reconciling the dichotomy between anti-inflammatory and pro-phagocytic mechanisms in mice.^141–143^ A recent study confirmed that Resolvin D3 can promote angiogenesis after SCI.^136^ Investigating into the metabolites or synthetic derivatives of Omega-3 fatty acids and their molecular pathways has yielded novel perspectives on potential pharmacological interventions.

Thus far, several molecules have been identified as key mediators of Omega-3 fatty acids transport and cellular uptake. Up-regulation of fatty acid-binding protein 5 (FABP5) following SCI is associated with alterations in long-chain polyunsaturated fatty acids metabolism.^94^ Administration of small interfering RNA targeting FABP5 hinders the Omega-3 fatty acids-mediated improvement in locomotor function.^94^ *FABP5*^*-/-*^ mice have been shown to exhibit reduced uptake of exogenous DHA by brain endothelial cells and cerebral capillaries, leading to decreased endogenous DHA levels in the brain parenchyma and subsequent cognitive deficits.^144^ CD36 facilitates esterification, enhancing fatty acids uptake without directly catalyzing transmembrane translocation of fatty acids.^145^ Fatty Acid Transport Protein 1, located in the cerebral microvascular basement membrane, catalyzes the translocation of DHA into the brain, increased membrane translocation thereby expediting the cerebral DHA replenishment^146^ (Table 2).

**Table 2:** Basic research on the utilization of Omega-3 fatty acids in the treatment of spinal cord injury

Strategy	Administration	Animal model	Effect	Mechanism	Related pathway	Year	Reference
Preventive Diet							
DHA 2.1μmol/mL diet	i) Preventive DHA-enriched diet ii) FABP5 siRNA intraspinal injection	Rat T10 contusion model	7dpi motor function recovery	FABP5 promotes cellular uptake, transport, and metabolism of DHA	-	2016	^94^
Omega-3 fatty acid 0.55% diet	Preventive 8 weeks Omega-3 fatty acid enriched diet	Rat T10 contusion model	-	i) Regulating metabolic homeostasis ii) Increasing antioxidant defenses	i) Glucose, Polar uncharged/hydrophobic amino acids↓ ii) β-alanine, Carnosine, Homocarnosine, Kynurenine↑	2014	^107^
DHA (12.82 g), EPA (6.91 g) per 100 g diet	Preventive 8 weeks Omega-3 fatty acid enriched diets	Rat T10 contusion model	2wpi-8wpi motor function recovery	Generating neuroprotective and restorative neurolipidomes	Metabolomics data analysis	2013	^262^
Post-Injury Diet							
DHA 400 mg/kg/d diet	DHA-enriched diet after injury	Mouse T12 compression model	2dpi-28dpi motor function recovery	i) Increasing neuron, neurofilament and oligodendrocyte survival ii) Reducing microglia/macrophage activation	-	2013	^88^
DHA 1.2% diet	DHA-enriched diet after injury	Rat thoracic region compression model	4dpi-42dpi motor function recovery	i) Increasing neuroprotection, ii) Neutralizing the clinical and biochemical effects of myelopathy	i) BDNF↑ ii) Syntaxin-3↑ iii) 4-HNE↓	2012	^123^
DHA 400 mg/kg/d diet	DHA-enriched diet after injury	Rat T12 compression model	-	i) Reducing damage to white matter ii) Increasing myelin, serotonergic fibers and axon neuroprotection	i) β-amyloid precursor protein in dorsal columns↑ ii) Cytoskeletal proteins↑	2010	^269^
DHA 400 mg/kg/d diet	DHA-enriched diet after injury	Rat T12 compression model	4dpi-42dpi motor function recovery	i) Reducing macrophage recruitment and inflammation ii) Increasing neuron and oligodendrocyte survival	lipid peroxidation, protein oxidation, RNA/DNA oxidation COX-2↓	2007	^87^
Oral gavage of Omega-3 fatty acids dissolved in saline							
Omega-3 fatty acids 50 mg/kg/d, 100 mg/kg/d	Oral administration for 30 d	Rat T12 ischemia-reperfusion model	-	i) Reducing oxidative stress ii)Reducing neurons apoptosis iii) Suppressing inflammation	i) TNFα, IL-6↓ ii) P53, Caspase-3, Bax, pro-NGF↓ iii) Bcl-2↑	2019	^85^
Intravenous Injection							
Omega-3 fatty acids 250 nmol/kg in a volume of 5 mL/kg	intrajugular injection every 24 h for 3 d	Rat T10 contusion model	7dpi motor function recovery	i) Suppressing oaf microgliosis and inflammation ii) Increasing numbers of oligodendrocytes and prevent demyelination	NLRP3/NLRP1-IL1b-IL18↓	2021	^84^
DHA 250 nmol/kg in a volume of 2.5 mL/kg	Tail vein injection 30 min post injury	Rat C4-C5 hemisection model	6dpi-20dpi motor function recovery	i) Sprouting of uninjured corticospinal and serotonergic fibers ii) Increasing synaptic plasticity	Synaptic vesicle protein, Synaptophysin, Synaptic active zone protein↑	2017	^117^
Rehabilitation	3w staircase training						
DHA 250 nmol/kg in a volume of 5 mL/kg	Tail vein injection 1 day post injuty	Rat C4-C5 hemisection model	7dpi-21dpi motor function recovery	Increasing neuroplasticity	i) miRNA-21↑ ii) PTEN, P-AKT↑	2015	^124^
DHA 250 mmol/kg	Tail vein injection 30 min post injury	Mouse T6-T7 compression model	4dpi-10dpi motor function recovery	i) Reducing inflammation and tissue injury ii) Reducing glia scar formation iii) Reducing apoptosis iiii) Reducing oxidative stress	i) Fas-L, Bax, Bcl-2↓ ii) TNF-α ↓ iii) GFAP↓ iiii) Nitrotyrosine↓	2014	^86^
DHA 500nmol/kg in a volume of 5 mL/kg	Tail vein injection 30 min post injury	Mouse T12 compression model	2dpi-28dpi motor function recovery	i) Increasing neuron, neurofilament and oligodendrocyte survival ii) Reducing microglia/macrophage activation	--	2013	^88^
DHA 250nmol/kg	Tail vein injection 30 min post injury	Rat T12 compression model	-	i) Reducing damage to white matter ii) Increasing myelin, serotonergic fibers and axon neuroprotection	i) β-amyloid precursor protein in dorsal columns↑ ii) Cytoskeletal proteins↑	2010	^269^
DHA 250 mmol/kg	Tail vein injection 30 min post injury	Rat T12 compression model	4dpi-42dpi motor function recovery	i) Reducing macrophage recruitment and inflammation ii) Increasin neuron and oligodendrocyte survival	lipid peroxidation, protein oxidation, RNA/DNA oxidation COX-2↓	2007	^87^
DHA 250 mmol/kg	Tail vein injection 30 min post injury	Rat T8 hemisection model	3dpi-42dpi motor function recovery	i) Decreasing lesion size ii) Suppressing apoptosis iii) Suppressing oxidation iiii) Increasing neuron and oligodendrocyte survival	-	2006	^106^
Others							
Fat-1 Transgenic moue	Preventive gene therapy	Mouse T12 compression model	19dpi-28dpi motor function recovery	i) Increasing neurons, non-phosphorylated neurofilaments and oligodendrocytes survival ii) Reducing microglia/macrophage activation and pro-inflammatory mediators	i) IL-8↓ ii) IL-1b,IL-6↓	2013	^92^
Fenretinide 5 mg/kg/d	Oral administration	Mouse T11 contusion model	7dpi-28dpi motor function recovery	i) Decreasing AA and increase DHA levels in serum ii) Suppressing inflammation and oxidation	i) TNFα, iNOS↓ ii) Nitrotyrosine 3-malonyldealdehyde↓	2010	^95^

## Therapeutic effects of omega-3 fatty acids on complications of spinal cord injury

For an extended period following spinal cord injury (SCI), patients are susceptible to various health complications, including neuropathic pain, osteoporosis, and obesity. These complications arise as a result of prolonged bed rest due to paralysis, hyperinflammatory states, and metabolic disorders.^4,147–150^ Omega-3 fatty acids are known to exhibit comprehensive effects on systemic diseases.^151,152^ Thus, it is hypothesized that Omega-3 fatty acids may play a role in alleviating complications in the chronic phase of SCI.

### Neuropathic pain

Neuropathic pain (NP) is a persistent and challenging complication of SCI that significantly impacts the patient’s quality of life.^148^ While traditional pain management strategies are often ineffective in managing NP,^153^ various studies in recent years have shown that Omega-3 fatty acids show potential in alleviating these symptoms. Figueroa et al.^154^ found that feeding rats an Omega-3 fatty acids-enriched diet for 8 weeks before SCI significantly reduced NP sensitivity. Functional neurometabolomic analysis revealed a significant dysregulation in the metabolism of endocannabinoids and related N-acyl ethanolamines (NAEs) at 8 weeks post-SCI. NAEs and endocannabinoids are bioactive lipids mediators that play crucial roles in the regulation of pain signaling pathways.^155^ They exert their effects primarily through cannabinoid receptors, which are involved in modulating neuropathic pain following spinal cord injury.^156^ Consumption of Omega-3 fatty acids led to a substantial accumulation of new NAE precursors and reduction in inositols, which are known biomarkers of chronic neuropathic pain. Omics analysis provided new evidence that Omega-3 fatty acids improve the anti-hyperalgesic phenotype at the metabolic level. This analgesic effect can also be attributed to decreased sprouting of calcitonin gene-related peptide (CGRP) nociceptive fibers and the downregulation of p38 MAPK in the dorsal horn.^154^ Additionally, the intrathecal administration of Resolvin for a period of 3 weeks after SCI in mice was found to prevent mechanical allodynia and heat hyperalgesia by inhibiting microgliosis and TNF-α release.^157^ At the same time, preclinical and clinical investigations have demonstrated that Omega-3 fatty acids can alleviate NP resulting from chronic sciatic nerve compression injury and diabetic peripheral neuropathy by activating the opioid system.^158–161^ In addition, some research suggests that Omega-3 fatty acids may have potential as analgesics. Omega-3 fatty acids and their derivatives can regulate pain-related Transient Receptor Potential (TRP) channels, such as TRPV1, TRPV3, TRPA1, and TRPM8.^162,163^ These channels play a role in pain treatment by modulating ion channel signaling. However, clinical data on the analgesic effects of Omega-3 fatty acids are still limited.^164^

### Osteoporosis

Osteoporosis is a systemic bone disease characterized by decreased bone mass and impaired bone microstructure, leading to bone fragility and increased fracture risk.^165^ Osteoporosis is one of the main complications among patients with SCI.^166^ Although the impact of SCI on bone vary depending on the skeletal site, sex, and age,^167^ most individuals with complete motor SCI develop significant bone loss or osteoporosis below the level of injury.^168^ Studies indicate that about 40% of patients with chronic SCI develop osteoporosis and fractures, facing twice the risk of those without SCI.^169^ The most common sites for fractures due to SCI-related osteoporosis are the proximal tibia, followed by the distal femur.^170^ Individuals with SCI typically exhibit rapid and severe bone density loss, significantly increasing their risk of developing osteoporosis.^171,172^ This bone loss occurs in two distinct phases. During the acute phase of SCI, both osteoblastic and osteoclastic activity initially increase, followed by a shift characterized by elevated osteoclastic activity and suppressed osteoblastic activity.^173,174^ This suppression of osteoblastic activity persists for several months post-injury before returning to pre-injury levels. In the chronic phase, the body exhibits sustained elevated osteoclastic activity and bone resorption.^175,176^

Neuron impairment in patients may lead to upregulation of receptor activator of NF-κB ligand (RANKL) and dysregulation of Wnt signaling in bones, thereby promoting bone resorption and inhibiting osteogenesis.^177^ In addition, the inflammation associated with SCI is one of the major pathological mechanisms leading to this complication.^177,178^ IL-1, IL-6, TNF-α, and prostaglandin E2 (PGE2) have been identified as stimulators of bone resorption.^179^

Over two decades ago, researchers confirmed that Omega-3 fatty acids, among various dietary fatty acids, can regulate osteogenic and osteoclastic functions while enhancing bone density in animal models.^180,181^ In vitro and in vivo studies prove that EPA can regulate inflammation-related factors, including PGE2, IL-6, and TNF-α, by reducing AA levels on cell membranes.^180^ Additionally, EPA can upregulate insulin-like growth factors 1 (IGF-1), regulate inflammation, inhibit bone resorption, and promote bone formation.^180,182^ Resolvins also have a significant protective effect on bone loss caused by inflammation.^183^

**Fig. 3:**
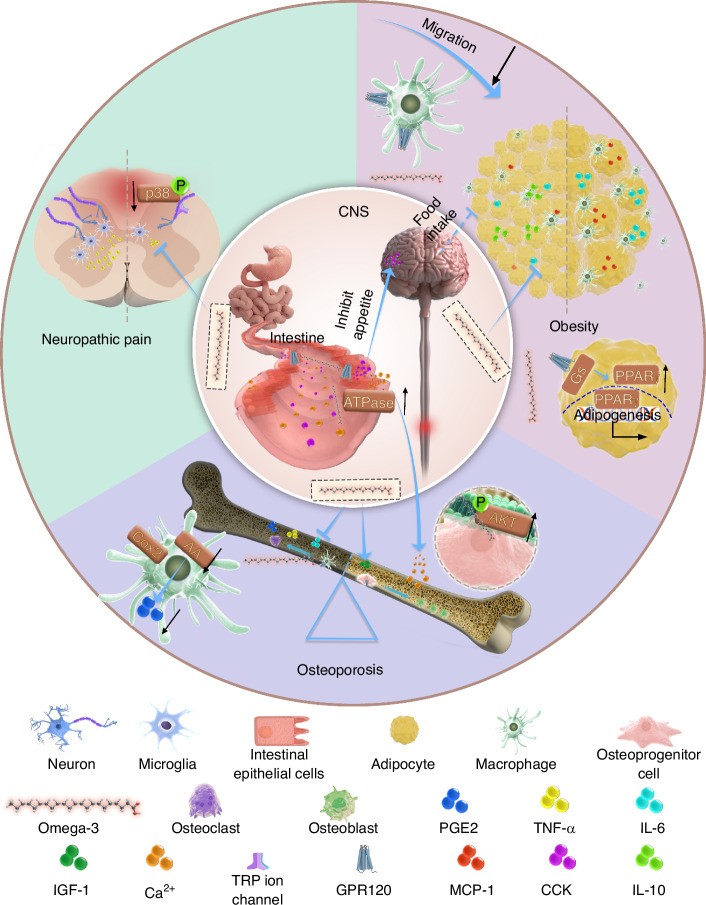
Effects of Omega-3 fatty acids on spinal cord injury complications. Post spinal cord injury, Omega-3 fatty acids attenuate pathological neuropathic pain by inhibiting pathological CGRP sensory neuron neogenesis, microgliosis, TNF-α release and p38-MAPK expression in the dorsal horn neuron of the spinal cord. Omega-3 fatty acids reduce bone resorption by inhibiting the secretion of pro-inflammatory relative molecules such as PGE2, TNFα, and IL-6 in bone. Omega-3 fatty acids promote intestinal uptake of calcium ions through activate ATPase and secretion of IGF-1 in bone, thus enhancing bone formation. Omega-3 fatty acids reduce macrophage infiltration by inhibiting the secretion of MCP-1 in adipose tissue and inhibiting the secretion of IL-6 and IL-10 thereby reducing the formation of adipose tissue. Also, GPR120 coupling with G_s_ can promote adipogenesis, while the lipid content within each adipocyte remains low, ultimately improving overall metabolic health. Omega-3 fatty acids activate GPR120 on intestinal epithelial cells, which enhances the uptake of CCK, and thus suppresses appetite

One of the primary physiological functions of Omega-3 fatty acids is maintaining cell membrane homeostasis, prompting researchers to investigate their impact on the plasma membrane.^184^ This investigation revealed that DHA-induced lipid profiles create more stable membrane microdomains and increase protein kinase B activity, thus promoting mesenchymal stem cell differentiation into osteoblasts.^184^ From the perspective of nutrition, Omega-3 fatty acids can enhance calcium absorption by activating ATPase activity in the duodenum, thus improving bone density.^185^ Omega-3 fatty acids may exert significant therapeutic effects on osteoporosis primarily through GPR120. Activation of GPR120 was shown to stimulate osteoblast differentiation and mineralization while inhibiting osteoclast differentiation and activity in a mice model of osteoporosis.^186,187^ The GPR120 signaling pathway can also inhibit osteoclast formation and bone resorption by inhibiting ROS production^188^ (Fig. 3).

Nevertheless, it should not be ignored that two randomized controlled trials on osteoporosis in SCI patients and healthy susceptible people did not produce evidence supporting the increase of bone density through dietary Omega-3 fatty acids supplementation.^189,190^ Further research is therefore required to elucidate the potential effects of Omega-3 fatty acids in preventing or mitigating osteoporosis in healthy individuals, as well as treating osteoporosis in patients with SCI, and determining the optimal dosage necessary to achieve the desired outcome.

### Obesity

Obesity—identified by body fat percentages exceeding 22% in males and 35% in females—is frequently observed among patients with SCI, with a prevalence rate of 85%.^191^ Obesity is a metabolic disorder characterized by an imbalance between energy intake and expenditure, leading to excessive fat accumulation.^192^ In individuals with SCI, this disorder is further exacerbated by inevitable muscle atrophy, impaired anabolic metabolism, and sympathetic dysfunction. These pathophysiological changes contribute to a marked reduction in overall metabolic rate through the spinal cord–adipose tissue axis and spinal cord-liver axis.^192–195^ Additionally, individuals with SCI often spend prolonged periods sitting and have notably reduced physical activity.^196^ These factors collectively disrupt energy balance, leading to alterations in body composition and resulting in neurogenic obesity. This neurogenic obesity places individuals at heightened risk for systemic inflammation, hyperglycemia, dyslipidemia, and hypertension.^197,198^ Previous studies have established that SCI patients have 8.5% to 13% more body fat per unit of body weight compared to able-bodied controls.^199–202^ Furthermore, one study found that myopenic obesity was prevalent in 41.9% of the sample population. Notably, appendicular lean mass index (ALMI) was lower in participants with complete motor injuries compared to those with incomplete motor injuries.^203^ Overall, these data suggest a remarkably high frequency of myopenic obesity in chronic SCI individuals.

Obesity further aggravates complications of SCI, including NP and type 2 diabetes.^204,205^ Therefore, prioritizing the management of obesity is essential for the management of other complications. A systematic review has evaluated different strategies for addressing obesity in patients with SCI, such as physical exercise, neuromuscular electric stimulation (NMES) or functional electric stimulation (FES), pharmacotherapy, surgery, and diet therapy.^206^ However, these approaches either produce additional adverse effects in individuals with SCI or yield minimal clinical benefits. Therefore, patients with SCI urgently need better treatment strategies with fewer side effects to control obesity.

Obesity is recognized as a chronic low-grade inflammatory condition.^207^ There is substantial evidence that Omega-3 fatty acids supplementation can help prevent obesity or further weight gain. Anti-inflammation is the most commonly mentioned mechanism.^204,208^ On the one hand, supplementation with DHA and EPA effectively attenuates adipose inflammation by reducing the production of pro-inflammatory cytokines, including monocyte chemotactic protein-1 (MCP-1), IL-6, and resistin, while simultaneously increasing the release of anti-inflammatory cytokines, such as adiponectin and IL-10 in mice adipose tissue.^209,210^ Additionally, in obese individuals, Omega-3 fatty acids reduce M1 macrophage infiltration, curb the generation of Omega-6-derived pro-inflammatory lipid mediators, and provide substrates for pro-resolutive lipid mediators.^211,212^

Meanwhile, GPR120 promotes adipogenesis through TULP3-dependent ciliary localization: under Omega-3 fatty acids stimulation, GPR120 enhances cAMP secretion, subsequently, activating EPAC signaling, CTCF-dependent chromatin remodeling, and transcriptional activation of PPARγ and CEBPα to initiate adipogenesis.^30^ Although GPR120 coupling with G_s_ can promote adipogenesis, particularly by increasing the number of adipocytes, the lipid content within each adipocyte remains low. This mechanism fosters hyperplastic expansion of white adipose tissue rather than ectopic fat deposition in other tissues, ultimately improving overall metabolic health. This finding provides a new explanation for the role of Omega-3 fatty acids in the treatment of obesity and their anti-diabetic effects.^30^

In addition to playing a role at the metabolic level, Omega-3 fatty acids can also help with weight loss by suppressing hunger^42,213^ and promoting postprandial satiety,^214,215^ thus reducing food intake (Fig. 3; Table 3).

**Table 3:** Basic research on the use of Omega-3 fatty acids for treating spinal cord injury-related complications

Complication type	Strategy	Administration	Model	Effect	Mechanism	Reference
Neuropathic pain	Omega-3 fatty acid enriched diet	Preventive 8w Omega-3 fatty acid–enriched diet	Rat T10 contusion model	Attenuation of thermal hyperalgesia at 4 wpi	i)Accumulation of new NAE precursors ii)Decreasing sprouting of CGRP nociceptive fibers	^154^
Neuropathic pain	Resolvin E1	Intrathecal injection 100 ng daily for 3 d pre- or post-SCI	L5 spinal nerve ligation model	Partially preventing the development of mechanical allodynia	i)Downregulation of Iba-1 ii)Blocking the release of Tnf-α of microglia	^157^
Neuropathic pain	2.3 g/kg and 4.6 g/kg of EPA 1.9 g/kg and 3.8 g/kg of DHA	Daily oral administration for 10 d after injury	Partial sciatic nerve ligation model	Preventing mechanical and thermal sensitization	i)Reducing Tnf-α level in spinal cord ii)Reducing ATF-3 expression in DRG cells	^285^
Neuropathic pain	0.36 g/kg and 0.72 g/kg Omega-3 fatty acids	Daily oral administration for 21 d after injury	Partial sciatic nerve ligation model	Reversing thermal hyperalgesia and reducing mechanical allodynia without neuroma formation	Restoring axonal density and morphology	^158^
Neuropathic pain	Fat-1 Transgenic moue	Preventative gene therapy	Formalin injection-induced inflammatory pain model	Attenuating pain sensitivity	i)Inhibiting microglial activation ii)Attenuating inducible nitric oxide synthase expression	^286^
Neuropathic pain	1 mg/kg EPA	Intravenous or intrathecal injection after injury	Chemotherapy- and inflammation-induced neuropathic pain model	Attenuating neuropathic and inflammatory pain	Inhibiting VNUT-mediated ATP uptake	^287^
Neuropathic pain	100, 250 or 500 nmol/kg DHA	Intravenous injection of DHA 30 min after median nerve injury	Median nerve chronic constriction injury model	Modulating development of behavioral hypersensitivity	i)Decreasing p-JNK and OX-42 levels ii)Diminishing the release of proinflammatory cytokines	^288^
Osteoporosis	Mimic DHA-enriched diets in vitro	20 μmol/L DHA cell culture environment	In vitro model of MSC differentiation into osteoblasts	Enhancing the osteogenic differentiation potential of MSCs	i)Remodeling lipidomic ii)Enhancing Akt activation in plasma membrane	^184^
Obesity	36 g/kg EPA	High saturated-fat diet for 6 weeks, followed by high saturated-fat EPA reversal for 5 weeks	High saturated-fat diet-induced obesity	Prevent and improve insulin resistance induced by High saturated-fat diet	i)Reducing adipose inflammation ii)Elevating markers of fatty acid oxidation	^26^

## Gpr120 as a novel GPCR target for spinal cord injury treatment

### GPCRs in spinal cord injury mechanisms and therapeutic potential

GPCRs constitute the largest family of membrane-bound receptors in the human genomes, playing pivotal roles in regulating various physiological and pathological processes.^216^ GPCRs exert significant influence on SCI, critically modulating secondary injury and repair processes. Studies have shown that many GPCRs are vital for regulating neuroinflammation, a hallmark of secondary injury after SCI, driven by cytokine, chemokine, and immune cell activation.^217^ CX3CR1, a microglia-enriched chemokine receptor activated by CX3CL1, plays a critical role in the regulation of neuroinflammation after SCI by regulating the interactions between neurons, microglia, and immune cells.^218,219^ In addition, protease activated receptor 1 (PAR1) and protease activated receptor 2 (PAR2) also play important roles in the inflammatory response after SCI.^220,221^ PAR1 knockout could improve motor recovery, decrease the proliferation of inflammatory cells, and reduce the levels of pro-inflammatory cytokines such as IL-1β and IL-6 in mice.^220^ Similarly, knockdown of the PAR2 gene also resulted in a significant improvement of motor recovery in SCI mice, as well as a reduction in the secretion of pro-inflammatory cytokines including IL-6, TNF-α and IL-1β.^221^ This suggests that PAR1 and PAR2 serve as novel drug targets to suppress neuroinflammation. The GPCR family further includes GPR34 and GPR55 as key inflammatory modulators.^222,223^ GPR34 knockout resulted in reduced levels of pro-inflammatory cytokine and inhibited the pro-inflammatory response of microglia in mice.^224^ GPR55 also acts as a key target in the regulation of neuroinflammation, and its agonist CID16020046 has a favorable anti-inflammatory effect by activating GPR55 and thereby mediating the JAK2/STAT3 pathway to alleviate neuroinflammation in rats.^225^ Adhesion GPCRs play diverse roles in neurogenesis and metabolic homeostasis.^226^ A defining feature of adhesion GPCRs is their large extracellular N-terminal fragment, which serves as an ideal target site for the development of therapeutic antibodies or allosteric modulators.^227^ This characteristic offers significant potential for designing highly effective anti-inflammatory treatments for SCI.^228^

Except inflammation, GPCRs are increasingly recognized as having therapeutic potential in NP.^229^ Studies show that GPR160 expression increases in the rodent dorsal horn of the spinal cord after SCI, and inhibiting GPR160 prevents and reverses NP in rodents without changing normal pain responses. Also, inhibition of GPR160 in the spinal cord attenuated sensory processing in the thalamus, a key relay station in the pain sensory discrimination pathway. These results identify GPR160 as a key factor and potential therapeutic target for NP.^230^ Sphingosine-1-phosphate receptor (S1PR) is also a therapeutic target for NP, and its agonist fingolimod (FTY720) attenuates nociception in preclinical pain models through either activation (agonism) or inhibition (functional antagonism) of S1PR.^231,232^ Other GPCRs that play important roles in NP such as GalR2, GPRC5B and GPR151, are also crucial for the development of drugs related to the treatment of NP.^233–235^ Intriguingly, Omega-3 fatty acids also exhibit dual therapeutic potential in modulating neuroinflammation and alleviating neuropathic pain, but the receptor on which they exert their effects remain unclear.

### Recognition pattern of Omega-3 fatty acids double bonds by GPR120

With seven transmembrane helices, GPR120 can activate multiple downstream effectors, including G proteins (such as G_s_, G_i/o_, G_q/11_) and arrestins (such as β-arrestin1, β-arrestin2).^236^

In a recent study, Mao et al. elucidated the signal transduction mechanism of GPR120 in detail: the study utilized single-particle cryo-electron microscopy to achieve a high-resolution structure of the GPR120-G protein complex with different ligands, including EPA, 9-hydroxystearic acid (9-HSA), oleic acid (OA), linoleic acid (LA), and synthetic compound ligand TUG891. They identified a series of aromatic residues that were able to individually recognize specific double C-C bonds present in unsaturated fatty acids. In particular, each of these aromatic residue arrays, such as Phenylalanine (F27N-term, F28N-term, F882.53, F1153.29, F2115.42, and F3037.35) and Tyrosine (W198ECL2, W2075.38, W277 6.48), recognizes one to three double bonds at specific positions of these unsaturated fatty acids. Further experimental verification revealed that these aromatic residues recognize double bonds through π:π interactions and maintain defined angles and distances, which in turn affect different downstream signal transduction.^39^

**Fig. 4:**
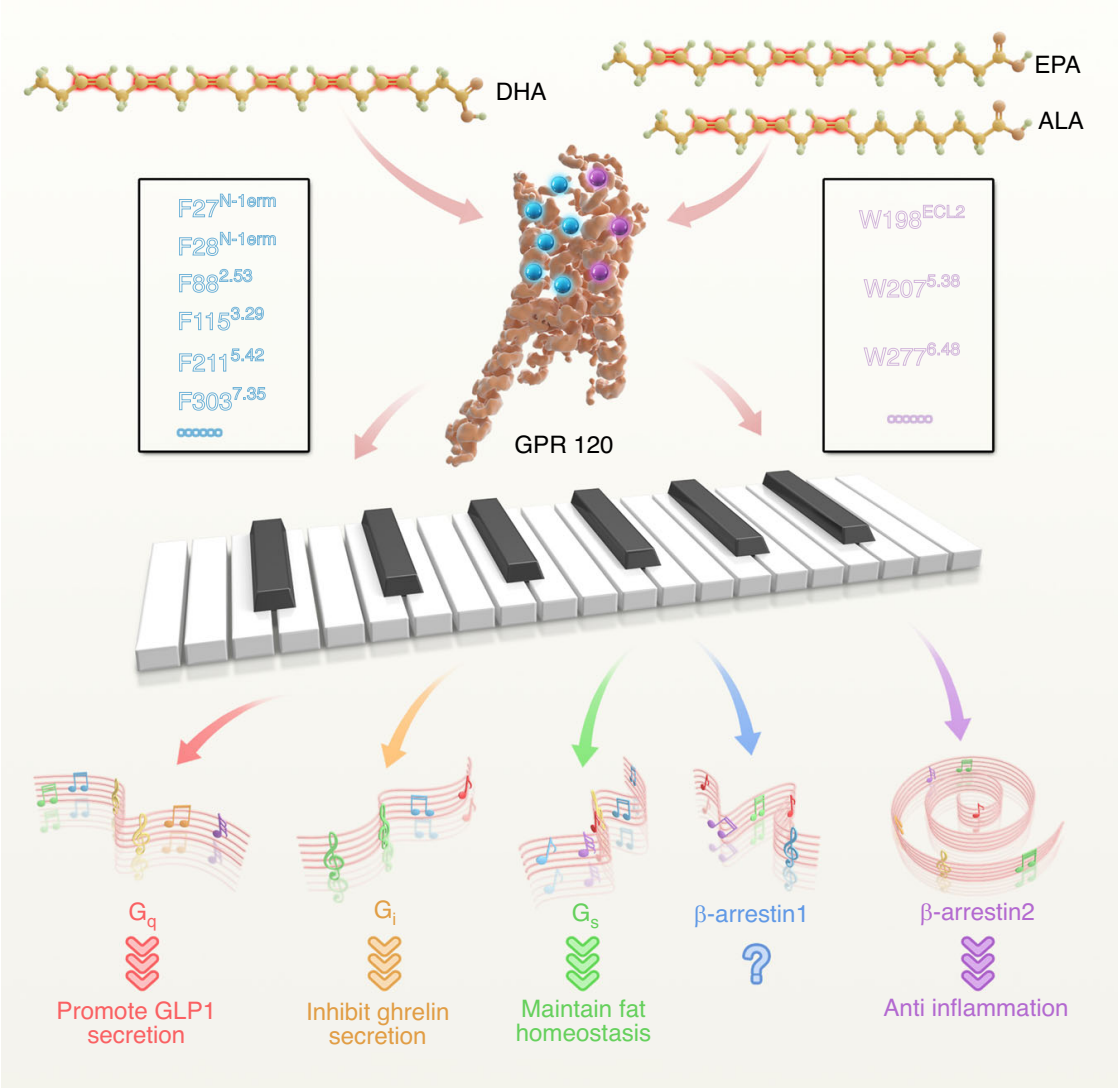
Overview of recognition mechanism and functions of Omega-3 fatty acids and GPR120. The free fatty acid receptor GPR120 specifically recognizes the C–C double bonds present in Omega-3 fatty acids, through π:π interactions between aromatic residues (Phenylalanine and Tyrosine) and double bonds. The interaction patterns of different C–C double bonds among various Omega-3 fatty acids within the ligand pocket of GPR120 are translated into different signaling outcomes via distinct propagating paths. The G_q_ pathway(red) of GPR120 stimulates GLP-1 secretion from intestinal L cells, while G_i_-mediated signaling(orange) is necessary for GPR120 to suppress Ghrelin secretion in stomach cells. GPR120’s G_s_(green) function is crucial for maintaining ciliary fat homeostasis. Additionally, GPR120’s anti-inflammatory effects are facilitated through its interaction with the β-arrestin2 pathway(purple). Blue arrow and notes suggest a possible role for β-arrestin1, whose function remains less defined. Blue sphere and letters on the left side of GPR120 represent the Phenylalanine. purple sphere and letters on the right side represent purple sphere and letters on the right side represent the Tyrosine

This discovery fills the gap in long-chain fatty acids recognition by G protein-coupled receptors. It revealed that the GPR120 has at least nine aromatic residues that selectively recognize the arrangement of double bonds at different positions in unsaturated fatty acids, converting them into distinct signal outputs to create a piano key-like encoding system. Consequently, the study proposes a “piano keyboard” model for the recognition of aromatic residues in GPR120 and double C-C bonds in unsaturated fatty acids. The “keyboard” is made up of a series of double bonds in the unsaturated fatty acids, while specific aromatic residues of GPR120 act as the “fingers”. When in contact with the “keyboard” of unsaturated fatty acids, these “fingers” produce distinct “tunes” to guide biological functions. Based on these findings, unsaturated fatty acids can be modeled as “keyboard models” with different double bond modifications at specific locations that selectively connect to preference signal transduction pathways. By understanding the structural features of unsaturated fatty acids recognized by GPR120, it may be possible to develop more high-selective and effective therapies for a range of diseases (Fig. 4).

In this study, the authors discovered that compared to the saturated fatty acids 9-HSA, EPA forms multiple additional π-π interactions via double bonds on its carbon chain with F27N-term, F1155.39, W1985.38, W2075.38, F2115.42 and F3037.35. Molecular biological experiments revealed that mutations F882.53 A, F2115.42 L and F3037.35 A exerted significantly greater impacts on the Gs signaling pathway mediated by GPR120 than on the Gi signaling pathway, indicating that these three residues recognizing double bonds iii, vi, and xii determine the Gs-biased signaling properties. These findings suggest that distinct π-π interactions between aromatic amino acids on the receptor and the double bonds of the Omega-3 fatty acids EPA drive GPR120-mediated activation of different downstream signaling pathways.^39^ Clarifying the structural basis of ligand-receptor interactions is critical for new drug development.^237–239^ This study will facilitate the development of high-selective GPR120 agonists and selectively exert their beneficial effects. In addition, by optimizing the specific action sites of ligands, the development of highly selective and high-affinity ligands will effectively reduce the dose of drugs, thereby reducing the occurrence of side reactions and improving the safety of drugs. The piano keyboard model elucidates the recognition pattern of Omega-3 fatty acids and GPR120 under molecular level, which will help us to further explore the function of GPR120 after SCI and develop high-selective and high-affinity drugs for SCI treatment.

### Molecular pathway and effects of Omega-3 fatty acids mediated by GPR120

Activation of GPR120 is known to engage various downstream effectors, including G proteins and arrestins, to exert diverse biological functions.^236^ For example, GPR120 facilitates the recruitment of G_q_ to the plasma membrane. These proteins then interact with phospholipase C-beta, initiating the hydrolysis of phosphatidylinositol 4,5-bisphosphate (PIP2) to produce inositol 1,4,5-trisphosphate (IP3) and diacylglycerol (DAG). The increased IP3 then triggers the release of Ca^2+^, which plays important roles in mediating hormone secretion and glucose uptake.^240,241^ Activation of the G_q_ pathway promotes glucagon-like peptide-1 (GLP-1) secretion from pancreatic islet cells and enhances insulin release, thereby reducing blood glucose levels.^242,243^

Another downstream effector of GPR120 is G_i_, which inhibits adenylate cyclase activation, resulting in decreased cyclic adenosine monophosphate (cAMP) levels. Moreover, activation of the G_i_ can lead to the stimulation of other signaling pathways, including the mitogen-activated protein kinase (MAPK) pathway, which can lead to the phosphorylation of a variety of downstream targets, including transcription factors and other signaling proteins.^236,241,244^ Some researchers found that activation of the G_i_ pathway can reduce the secretion of ghrelin by enteroendocrine cells.^245^ Other studies have indicated that GPR120 couples with G_i_ proteins in delta cells, resulting in reduced somatostatin secretion under glucose stimulation conditions.^246^

GPR120 couples with G_s_ to stimulate adenylate cyclase activity and elevate intracellular cAMP levels. This activation triggers protein kinase A (PKA), leading to the phosphorylation of several downstream targets such as transcription factors, ion channels, and other signaling proteins.^28,241,247^

GPR120 can also couple with β-arrestin2: following G protein activation, GPCR kinases phosphorylate the GPCR cytoplasmic domain, creating phosphorylation motifs that recruit β-arrestin2, facilitating complex internalization or initiating separate signaling cascades that mediate distinct biological effects.^241^ The GPR120 pathway mediated by β-arrestin2 is crucial for the anti-inflammatory effects of Omega-3 fatty acids: activated β-arrestin2 can prevent inflammation-driven diseases by inhibiting NLRP3 inflammasome assembly.^29^ Moreover, the anti-inflammatory effects of β-arrestin2 are significantly beneficial for maintaining pancreatic stability and improving glucose metabolism.^248^ In addition, β-arrestin2 can interact with transforming growth factor-β-activated kinase 1 (TAK1) and transforming growth factor-β-activated binding protein 1 (TAB1), disrupting pro-inflammatory gene expression mediated by TAK1-TAB1, thus improving inflammatory neuropathy in microglia.^249,250^

**Fig. 5:**
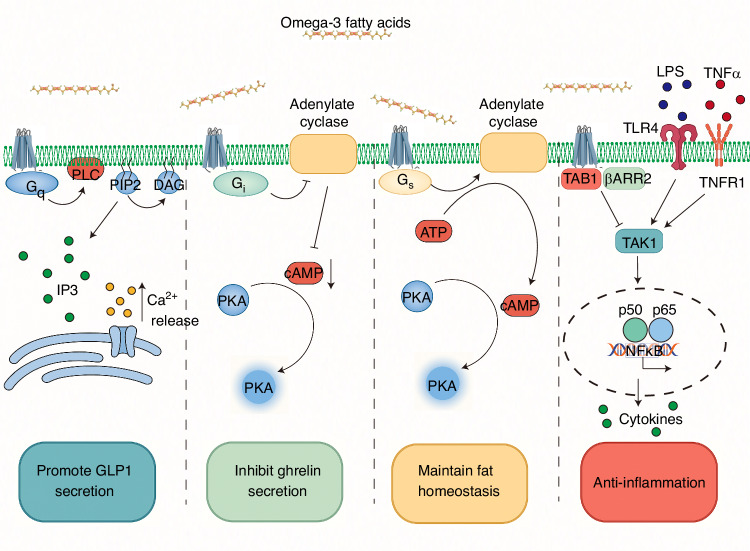
Molecular pathway and effects of Omega-3 fatty acid mediated by GPR120. GPR120 couples with multiple G proteins (G_q_, G_i_, G_s_) and β-arrestin2(βARR2) to regulate diverse physiological processes: (1)G_q_ activation recruits phospholipase C-beta (PLC-β), hydrolyzing phosphatidylinositol 4,5-bisphosphate (PIP2) into inositol 1,4,5-trisphosphate (IP3) and diacylglycerol (DAG). IP3 triggers calcium ion (Ca²⁺) release from the endoplasmic reticulum, stimulating the secretion of glucagon-like peptide-1 (GLP-1). (2)G_i_ activation inhibits adenylate cyclase activity, leading to decreased cyclic adenosine monophosphate (cAMP) levels and inhibition of ghrelin secretion. (3)G_s_ activation enhances adenylate cyclase activity, increasing cAMP and activating PKA to maintain fat homeostasis. (4)βARR2 exerts anti-inflammatory effects by blocking pro-inflammatory gene expression mediated by the TAK1-TAB1 complex (such as TLR4/TNFR1 pathways) and suppressing NLRP3 inflammasome assembly

Due to its extensive interactions with various downstream effectors, GPR120 has potential therapeutic implications for multiple diseases, including type 2 diabetes, obesity, and inflammation-driven diseases.^55,251^ Dysfunction of GPR120 may lead to obesity in mice and humans.^252^ Furthermore, a class of endogenous mammalian lipids (palmitic acid esters of hydroxy stearic acids), which have anti-diabetic and anti-inflammatory effects, was recently discovered, and they can enhance insulin-stimulated glucose uptake through GPR120.^253^ Moreover, GPR120 agonists can improve glucose tolerance, reduce hyperinsulinemia, increase insulin sensitivity, and improve islet inflammation in high-fat diet mice.^248^ Another research indicates that GPR120 is associated with the pathogenesis of both NAFLD and ALD.^254,255^ Activation of GPR120 has been shown to improve hepatic lipid metabolism and attenuate liver inflammation.^256^ These effects may contribute to the modulation of spinal cord injury pathology through the spinal cord–liver axis (Fig. 5).

Apart from its critical role as a drug target in glucose and lipid metabolism disorders, GPR120 functional imbalances can also contribute to the development of various other diseases. GPR120 has been implicated in the regulation of bone metabolism and the development of osteoporosis: activation of GPR120 has been shown to stimulate osteoblast differentiation and mineralization while inhibiting osteoclast differentiation and activity in animal models of osteoporosis.^186,187^ Furthermore, the GPR120 signaling pathway can inhibit osteoclast formation and resorption by suppressing reactive oxygen species (ROS) production.^188^

In summary, GPR120 significantly influences various metabolic diseases primarily through its anti-inflammatory functions. Research into the molecular structure and signal transduction mechanism of GPR120 can provide fresh insights into the prevention and treatment of various metabolic diseases. GPR120 is expressed in spinal cord tissue, and its expression levels are increased after SCI.^54^ However, the precise role of GPR120 in the onset and progression of SCI remains poorly understood. Therefore, a full understanding of the downstream molecular pathway of GPR120 will help us to clarify the function of GPR120 in the occurrence and development of SCI. Targeting relevant molecular pathways through activation or inhibition of GPR120 signaling may represent a novel therapeutic approach for SCI treatment (Table 4).

**Table 4:** Biological functions of GPR120 mediated by different downstream effectors

Effector	Metabolic process	Host cell	Signaling pathway	Biological functions	Related diseases	Year	Reference
G_q_	Glucose metabolism	L-cell	i) PIP2 → IP3 + DAG ii) Ca^2+^↑	Stimulating the secretion of GLP-1, increasing insulin release and reducing blood glucose levels	Diabetes	2008 2023	^242,243^
G_i_	Ghrelin secretion	Ghrelin cell	cAMP↓	Reducing the secretion of ghrelin	Anorexia	2013	^245^
G_i_	Somatostatin secretion	Pancreatic delta cell	cAMP↓	Reducing rates of somatostatin secretion under conditions of glucose stimulation.	Digestive system dysfunction	2014	^246^
G_s_	Lipid metabolism	Preadipocyte	i) cAMP↑ ii) CTCF↑ iii) PPARγ ↑ CEBPα ↑	Promoting adipogenesis	Obesity	2019	^30^
β-arrestin2	Inflammation	Macrophage	NLRP3↓	Preventing NLRP3 inflammasome dependent inflammation and metabolic disorder	Inflammation-driven diseases	2013	^29^
β-arrestin2	Glucose metabolism	Pancreatic cell	Unclear	Maintaining pancreatic stability and improve glucose metabolism	Diabetes	2022	^248^
β-arrestin2	Inflammation	Microglia	disrupting the TAB1-TAK1 interaction	Treating microglial inflammatory neuropathologies	Depression	2014 2014	^249,250^
Unclear	Lipid metabolism (PAHSAs)	Adipocyte	Unclear	Lowering ambient glycemia, improving glucose tolerance, stimulating GLP-1 and insulin secretion	Diabetes	2014	^253^
Unclear	Liver metabolism	Liver cell	i) cAMP↑ ii) Ca^2+^/CaMKKβ/AMPK signaling	Decreasing lipogenesis and lipid mobilization, reducing hepatic inflammation and oxidative stress	NAFLD and ALD	2019	^256^
G_s_	Bone metabolism	Osteoblast	Wnt/β-catenin signaling	Stimulating osteoblast differentiation and mineralization	Osteoporosis	2016 2019	^186,187^
β-arrestin2	Bone metabolism	Osteoclast	IKKβ signaling	Inhibiting osteoclast differentiation and activity	Osteoporosis	2016 2019	^186,187^

### Future perspectives for the clinical application of Omega-3 fatty acids

In contemporary medical practice, a comprehensive therapeutic approach has emerged, integrating diverse drug targets and surgical interventions, as well as strategies to enhance the body’s microenvironment and organ function. Ideally, these effects can be achieved by administering a single class of drugs capable of targeting multiple receptors.^257–261^ Omega-3 fatty acids—owing to their multifaceted properties which include anti-inflammatory, anti-oxidative, cell membrane protective, anti-apoptotic, and neurotrophic effects—demonstrate significant potential for modifying the microenvironment of SCI alongside the treatment and prevention of complications^85,86,129,262^ (Figs. 1, 2).

The administration route of Omega-3 fatty acids has been extensively investigated in numerous studies.^263–265^ Currently, Omega-3 fatty acids can be administered during the acute and chronic phases of SCI through oral supplementation and intravenous injection, respectively.^84,88^ Because of the necessity of prolonged oral intake to achieve Omega-3 fatty acids enrichment in cells and subsequent protective effects, one to three months of advance administration would be required.^266^ Moreover, given the practical difficulties of achieving the necessary high concentration by dietary intake alone, this approach is unlikely to be feasible in clinical settings.^267^ For acute administration, DHA and EPA are typically delivered intravenously 30 min after SCI.^86,88,117,268,269^ This acute intervention can rapidly target mechanisms active in the early stages of SCI, while dietary Omega-3 fatty acids may support the repair process.^266^ Accordingly, a combined strategy of intravenous Omega-3 fatty acid injection and oral supplementation should be considered in future clinical trial designs to provide ongoing protection following SCI.^266^ This perspective has been substantiated: a diet enriched with Omega-3 fatty acids, designed to mimic daily consumption, yielded therapeutic effects comparable to those of intravenous injection,^88^ while concurrent oral and intravenous administration resulted in a more pronounced therapeutic impact on SCI.^269^ Although several studies in animal models have reported the therapeutic effects of Omega-3 fatty acids on SCI, the optimal dose required to achieve maximum benefit remains uncertain.^128,267^

Dose optimization remains a critical challenge in studies investigating Omega-3 fatty acids administration. Unlike routine dietary consumption for disease prevention, SCI treatment necessitates higher doses that are not yet standardized for clinical use.^72,270,271^ High-dose administration is required to sufficiently activate GPR120 and achieve the desired therapeutic outcomes.^38,39^ Therefore, developing cost-effective and readily available alternatives to high-affinity fish oil represents an important step toward clinical application. Moreover, the development of GPR120 agonists and modulators of its downstream effectors has driven the search for effective Omega-3 fatty acid substitutes that could serve as future drug candidates for SCI treatment.^272^

## Conclusion

Over the past two decades, numerous studies have documented the therapeutic potential of Omega-3 fatty acids for SCI treatment. Omega-3 fatty acids significantly enhance the inflammatory, inhibitory, and nutritional microenvironment after SCI, thereby facilitating the repair of compromised neural circuits. Their cell membrane modification function also suggests avenues for identification and development of novel therapeutic targets for SCI. In addition to their neuroprotective effects, Omega-3 fatty acids are instrumental in preventing and treating various complications following SCI, including obesity, neuropathic pain, and osteoporosis. This multifaceted action broadens the scope of Omega-3 fatty acids’ comprehensive therapeutic effects on SCI.

Furthermore, the recently elucidated signal transduction mechanism underpinning the interaction between GPR120 and Omega-3 fatty acids offers a strong theoretical basis for designing highly selective, high-affinity drugs that target this receptor. Future drug development for SCI should explore activating different downstream effectors of GPR120 to enhance efficacy, reduce dosage, and mitigate side effects. Based on the interaction between Omega-3 fatty acids and GPR120, the development of fish oil substitutes with improved clinical significance may pave the way for more comprehensive and targeted SCI treatment.
